# Application of machine learning to the identification of joint degrees of freedom involved in abnormal movement during upper limb prosthesis use

**DOI:** 10.1371/journal.pone.0246795

**Published:** 2021-02-11

**Authors:** Sophie L. Wang, Conor Bloomer, Gene Civillico, Kimberly Kontson

**Affiliations:** 1 Division of Biomedical Physics, Office of Science and Engineering Laboratories, Center for Devices and Radiological Health, Food and Drug Administration, Silver Spring, Maryland, United States of America; 2 Department of Bioengineering, University of Maryland, College Park, Maryland, United States of America; 3 Office of the National Institutes of Health Director, National Institutes of Health, Bethesda, Maryland, United States of America; West Park Healthcare Centre, CANADA

## Abstract

To evaluate movement quality of upper limb (UL) prosthesis users, performance-based outcome measures have been developed that examine the normalcy of movement as compared to a person with a sound, intact hand. However, the broad definition of “normal movement” and the subjective nature of scoring can make it difficult to know which areas of the body to evaluate, and the expected magnitude of deviation from normative movement. To provide a more robust approach to characterizing movement differences, the goals of this work are to identify degrees of freedom (DOFs) that will inform abnormal movement for several tasks using unsupervised machine learning (clustering methods) and elucidate the variations in movement approach across two upper-limb prosthesis devices with varying DOFs as compared to healthy controls. 24 participants with no UL disability or impairment were recruited for this study and trained on the use of a body-powered bypass (n = 6) or the DEKA limb bypass (n = 6) prosthetic devices or included as normative controls. 3D motion capture data were collected from all participants as they performed the Jebsen-Taylor Hand Function Test (JHFT) and targeted Box and Blocks Test (tBBT). Range of Motion, peak angle, angular path length, mean angle, peak angular velocity, and number of zero crossings were calculated from joint angle data for the right/left elbows, right/left shoulders, torso, and neck and fed into a K-means clustering algorithm. Results show right shoulder and torso DOFs to be most informative in distinguishing between bypass user and norm group movement. The JHFT page turning task and the seated tBBT elicit movements from bypass users that are most distinctive from the norm group. Results can be used to inform the development of movement quality scoring methodology for UL performance-based outcome measures. Identifying tasks across two different devices with known variations in movement can inform the best tasks to perform in a rehabilitation setting that challenge the prosthesis user’s ability to achieve normative movement.

## Introduction

The loss of distal degrees of freedom (DOFs) in the arms and hands disrupts the performance of most activities of daily living [1]. Between the increased workload of the remaining limb and the altered joint kinematics that result from compensating for the loss of DOFs [2–4], individuals with upper limb loss are at greater risk for musculoskeletal pain and overuse injuries [1,5–7]. Understanding this risk, researchers and clinical groups are developing new performance-based outcome measures that incorporate subjective evaluation of how a task is being completed by asking the administrator to compare the movements of the prosthesis user to those of a person with a sound, intact upper limb [8,9].

Although these scoring approaches provide more insight into the functional abilities of individuals using upper limb prosthesis technology, the broad definition of “normal movement” and the subjective nature of the scoring can make it difficult to know which areas of the body to focus on evaluating, and the magnitude of the deviation from the normative movement ranges. There have been several studies utilizing quantitative motion capture to examine movement quality for the upper limb prosthesis user population [2–4,10–13], but these studies tend to isolate single features of motion and make narrowly-defined comparisons to non-disabled individuals. Additionally, these studies vary greatly in the kinematic parameters analyzed–from joint angles and range of motion [2–4,12–14] to velocity changes [11,15], rendering trend interpretations and cross study conclusions challenging.

Furthermore, these evaluations of movement are typically completed for a single terminal device at a time, making the effects of varying prosthetic technologies unclear. Consider two available technologies for this clinical population: the voluntary open body-powered hook controlled through body movements and the DEKA arm controlled with inertial measurement units attached to the feet [16–19]. With different prosthetic components and technology that provide different DOFs of control, one might anticipate kinematic differences in response to the available DOFs and control mechanism [20]. However, it is not yet clear where in the body and during what types of tasks these kinematic differences occur relative to normal movement and whether administrators should focus on certain areas of the body based on the type of prosthetic technology being used.

The identification of upper body movements as normal or not normal can easily be characterized as a binary classification problem. If upper body movement can be quantified into discrete features that describe the kinematic trajectories for a given DOF, clustering analysis can be used to systematically determine differences between two groups. Cluster analysis allows for the application of a global measure of separation (e.g. Euclidian distance) over an entire set of characteristics that describe movement instead of performing several individual comparisons for a single discrete movement characteristic (e.g. range of motion) that may be over- or underestimating differences between two groups, and require additional interpretation and subjective weighting on importance, especially with larger numbers of characteristics (or features) to be compared between groups. Individual comparisons (e.g. t-tests, ANOVAs, etc.) have their place in the identification of movements as normal or abnormal, but unsupervised clustering approaches provide an additional level of robustness by analyzing data without *a priori* knowledge of group characteristics [21]. The application of clustering analysis to identify movement differences between groups is not novel. Some studies have used input from inertial measurement units and accelerometers to classify different upper limb movements [22,23] or different gait patterns [24]. Other studies have applied clustering analysis and other machine learning techniques to motion capture data to classify different full body actions in healthy individuals [25] or severity of crouch gait in children with cerebral palsy [26]. Electromyography data of the abdominal and erector spinae muscles has also been used as input to clustering algorithms to classify patterns of muscle activity during gait in healthy controls [27]. While a couple of clustering studies have focused on the upper limb prosthesis user population, the application of clustering analysis to compare movement of individuals using multiple prosthetic devices to the movements of non-disabled individuals has not been done.

Based on the gaps described above, the goals of this work are to 1) identify DOFs that will inform abnormal movement for several tasks using unsupervised machine learning (clustering methods) and 2) elucidate the variations in movement approach across two upper-limb prosthesis devices with varying DOFs as compared to non-disabled controls. Motion capture data from non-disabled controls and individuals using two upper limb bypass prosthesis devices (body-powered hook and DEKA arm) will be used as input into a K-means clustering algorithm. The body-powered hook was chosen as a commonly used upper limb prosthesis terminal device [28–33], and the DEKA arm was chosen as a state-of-the-art terminal device with novel control mechanisms and powered DOFs [16,17,20,34]. By identifying DOFs involved in abnormal motion elicited through prosthesis device use, more targeted instructions in the performance-based outcome measures seeking to evaluate movement quality can be provided. Additionally, the results of the work can provide cross-device guidance for the evaluation of joints and movements typically involved in abnormal motion and insight into the differences in movement elicited by two different types of upper limb prosthesis devices. Lastly, identifying tasks with known variations in movement for specific device types can help inform the best tasks to perform in a rehabilitation setting that challenge the prosthesis user’s ability to achieve normative movement.

## Methods

### Participants

A total of 24 non-disabled participants with no upper limb disability or impairment and no prior prosthesis experience were included in this study. The study was approved by the FDA IRB (Protocol 16–071). All participants provided written informed consent prior to participating in the study. Each participant performed a set of standardized tasks under one of several conditions: Normative (Norm), Body Powered Bypass (BP Bypass), or DEKA Bypass (DK Bypass). To avoid confounding effects of the same individual being trained on two different prosthetic devices and to be mindful of participants’ time commitment to the study, each participant only participated in one condition.

**Fig 1:**
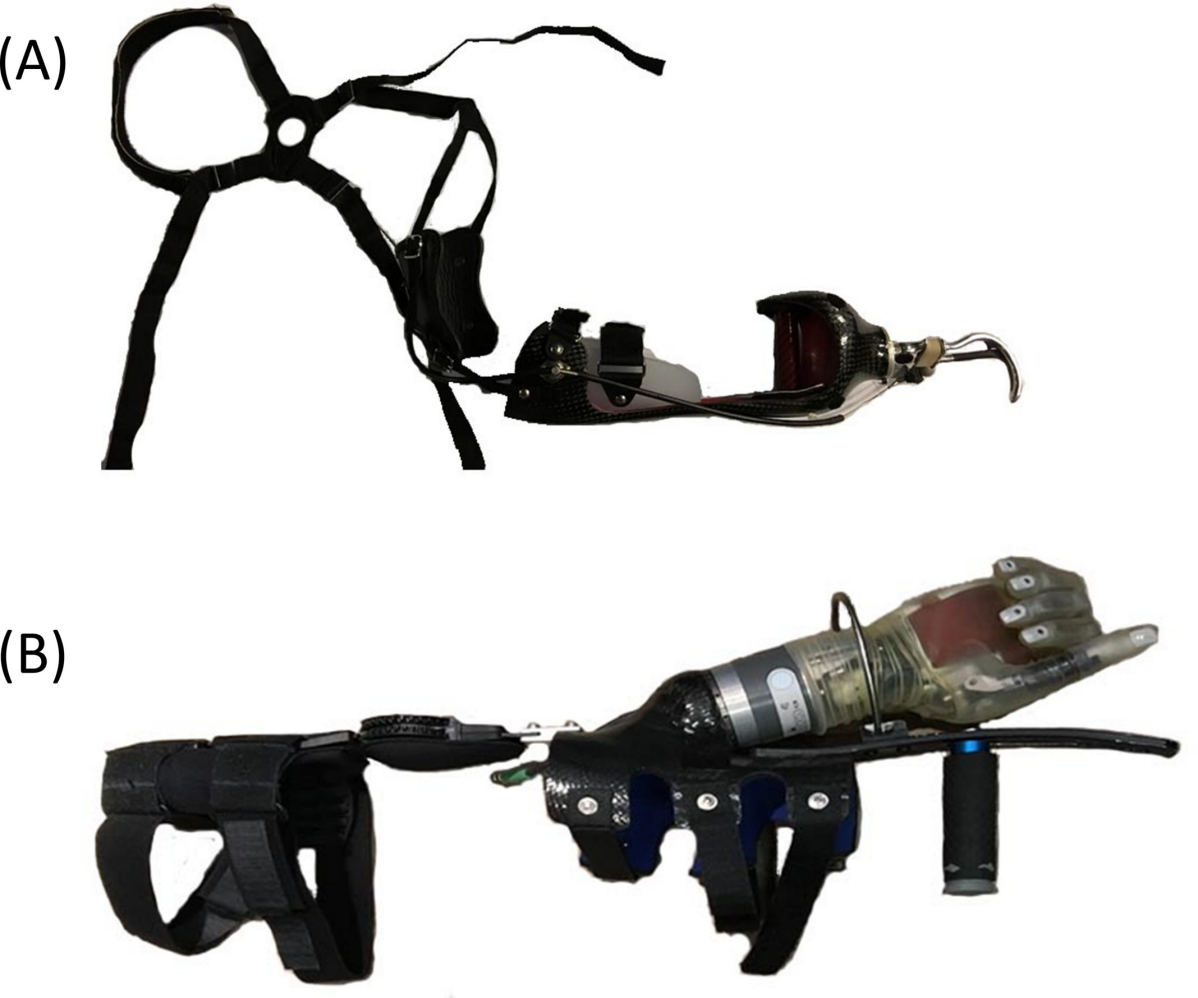
Upper limb prosthesis bypass devices. A) Body Powered Bypass device, right side configuration with body-powered voluntary open Hosmer 5x split hook terminal device. B) DEKA Bypass device, right-hand radial configuration with powered wrist.

In the bypass conditions, adaptive bypass devices allowed trained non-disabled participants to use upper limb prosthesis terminal devices (Fig 1A and 1B). Bypass devices are a well-accepted approach to assess skill acquisition, training efficacy, and device use performance [29,30,35–37]. The use of bypass devices was chosen in this study to isolate the influence of the upper limb terminal device on motion [38,39], as prosthesis users vary in terms of favored terminal devices, device training, and usage experience. Recruitment of individuals using the DEKA arm is also difficult since very few individuals throughout the country are trained on and using this device. In accordance with the protocol of Bloomer et al. [38], participants were trained for 20 hours on each bypass prosthetic device. Recommendations on training length for actual upper limb prosthesis users vary from 5 hours to several months [40,41], thus 20 hours of training is a reasonable approximation to training that would be received in a clinic. Further details on the participants and the devices within each of these condition groups are provided below.

#### Normative (Norm) condition

No devices were used in the Norm condition, and no training was provided. A convenience sample of 12 participants performed each task under the Norm condition (four females, eight males; mean age 26.91 ± 9.84 years). 11 of the 12 participants self-reported right-hand dominance, and all participants performed the standard tasks with the right hand. It was determined through examination of data distributions that left-hand and right-hand dominant individuals did not consistently differ in joint movement during tasks. Therefore, in order to maintain a higher sample size, left-handed individuals were included in the analysis.

The 12 participants were randomly assigned to two separate groups of six to serve as controls for the 6 BP Bypass and the 6 DK Bypass participants. The randomization was performed ten times to examine the effects of the variance in the normative condition. Further details of the randomization will be provided in the K-means clustering section below. None of the participants included in the Norm condition participated in any of the bypass conditions.

#### Body-Powered (BP) Bypass condition

In the BP Bypass condition (Fig 1A), a forearm brace adaptor with a perpendicular handlebar allowed the use of a body-powered voluntary open Hosmer 5x split hook terminal device, with manual locking wrist rotation, set in-line with the length of the forearm. On average, the length of the bypass prosthesis was 9.25 ± 0.64 cm longer than the intact limb. The body-powered bypass device was provided by Arm Dynamics (Dallas, TX). The in-line terminal device configuration was chosen due to the mechanical and kinematic requirements of the cable actuation with the figure eight harness.

A convenience sample of 6 participants (3 females, 3 males; mean age 28.16 ± 2.67 years) were trained with the BP Bypass device. All participants self-reported right-hand dominance. During standard task performance, the BP Bypass was set to a right-side configuration for all participants.

#### DEKA (DK) Bypass condition

For the DK Bypass condition (Fig 1B), a forearm brace adaptor provided by Next Step Bionics (Manchester, NH) with a perpendicular handlebar allowed the use the right-hand radial configuration motorized DEKA device with powered wrist. Just as the device is controlled by individuals with upper limb loss, non-disabled participants used inertial measurement units attached to the dorsum of the foot to control grip changes, opening and closing of the device, and wrist rotation. This terminal device was mounted to the forearm adaptor with a medial offset of 10° from the participant’s forearm.

A convenience sample of 6 participants (2 females, 4 males; mean age 27.67 ± 7.13 years) were trained with the DK Bypass device. 5 participants self-reported right-hand dominance, 1 participant self-reported left-hand dominance. All participants performed standard tasks with the right-hand radial configuration DK Bypass. It was determined through examination of data distributions that left-hand and right-hand dominant individuals did not consistently differ in joint movement during tasks. Therefore, in order to maintain a higher sample size, left-handed individuals were included in the analysis.

### Functional tasks

Participants performed two outcome measures: the Jebsen-Taylor Hand Function test (JHFT) and the targeted Box and Blocks Test (tBBT) [42,43]. These tasks were chosen as ones that would elicit a wide range of movements representative of those performed during activities of daily living. The JHFT consists of seven activities of daily living (ADL) tasks performed in a seated position: 1) Writing, 2) Page Turning, 3) Picking Up Small Objects, 4) Simulated Feeding, 5) Stacking Checkers, 6) Moving Large Light Objects and 7) Moving Large Heavy Objects [42]. A standard template was used to place each object for a given task in the same location for each participant. The tBBT involves the controlled transport of 16 blocks arranged in a 4 by 4 array from one side of a divided box to the other side [43]. This task was performed in both a standing position and a seated position [43]. Participants performed two trials of each task.

### Motion analysis

An eight-camera passive marker Vicon^TM^ motion analysis system was used to acquire and pre-process motion data (VICON, Oxford, UK). The Bonita B10 motion capture cameras were set to a sampling rate of 100Hz. Prior to each data collection session, the motion analysis system was calibrated according to manufacturer guidelines. Twenty-seven retro-reflective markers were placed on the upper body of each participant at the bony anatomical landmarks of the upper body in accordance with the Vicon Upper-Body Plug-In-Gait body model documentation (“Upper body modeling with Plug-in Gait,” 2019). Briefly, head markers were placed on the right/left temple and right/left back of the head; torso markers were placed on the spinous process of C7 and T10 vertebrae, right scapula, xiphoid process, and sternal notch; arm markers were placed on the acromio-clavicular joint, lateral surface of upper arm, lateral epicondyle of the elbow joint, lateral surface of lower arm, medial and lateral sides of the wrist joint, and on the third metacarpal; hip markers were placed on the right/left anterior superior iliac torso and right/left posterior superior iliac torso. The Plug-In-Gait upper body model was then calibrated to the dimensions of the participant to create the wrist, forearm, upper arm, head, neck, torso, and pelvic model segments.

### Data analysis

Joint angles were calculated from the Vicon upper body model using YXZ Euler angles derived from relative orientation comparisons of two segments (VICON Plug-In-Gait, Oxford, UK). Details on the calculation of kinematic parameters and model validation can be found in [44–46] The DOFs analyzed in this study include right and left elbow flexion/extension; right and left shoulder flexion/extension, abduction/adduction, and internal/external rotation; torso flexion, lateral flexion, and rotation; and neck flexion, lateral flexion, and rotation. Although the tasks performed were not bi-manual, we included DOFs at the left shoulder given the high incidence of musculoskeletal pain and injury occurring at the shoulder and on the contralateral limb for this clinical population [1]. The left elbow DOF was included to provide a complete understanding of the upper body/arm movement differences.

The angle data were then filtered using a 4^th^ order, zero lag, low pass Butterworth filter at 6 Hz. Data from each trial were further segmented into segments, with the number of segments depending on the number of objects manipulated in a given task. For example, the JHFT Task 2 -Page turning required the participant to turn over five notecards, which resulted in five segments. For all tasks, segment start was defined as approximately 10 frames before a participant contacted an object. Segment end was defined as approximately 10 frames after a participant let go of an object. A standard template was used to place each object for a given task in the same location for each participant, but the objects themselves may be distributed across the front of the participant or to the side of the participant depending on the task. To adequately compare kinematic trajectories across participants within a given condition, the analysis was limited to the last segment (i.e. object manipulated) within each trial.

### K-means clustering

K-means clustering was selected as the unsupervised machined learning method for this study due to the binary nature of the classification problem (i.e. Norm vs a Bypass), and the simplicity of the algorithm’s decision-making approach. Specific parameters, or features, of the joint angle trajectories were calculated to characterize the movement and serve as input into the K-means clustering algorithm. Given the current lack of consensus on the definition of movement quality as it pertains to evaluation of upper limb prosthesis users [47], several features describing different aspects of movement were selected for this analysis: peak angle, range of motion (RoM), joint trajectory path distance (PDist), mean joint angle, peak angle velocity, and number of zero crossings. The peak angle was calculated as the maximum measured joint angle value. The RoM was calculated as the difference between the maximum angle and the minimum angle value. The PDist was calculated according to Eq 1, wherein the absolute difference between each joint angle component sample point was calculated and summed across all samples taken (1 to *n* points) for a given DOF. For this metric, all trajectories were interpolated to be the same array length *n*, so no additional normalization to PDist was done.

$$P_{dist}=\sum_{1}^{n}\left|\left(x_{n+1}-x_{n}\right)\right|$$ (1)

Mean angle was calculated as the average of the joint angle over the task segment. The maximum value of the derivative of each joint angle served as the peak angle velocity. Smoothness was measured from zero crossing, where zero crossing is defined as the number of times angle velocity crossed zero [48,49]. The peak angle and RoM were selected as clinically accepted, accessible, and interpretable features of movement [4,11,42,43,50–55] and represent the movement envelope within which an individual performs a given task. PDist was selected as an additional summary metric to characterize movements as it has been previously used in the literature to examine compensatory motions in individuals with upper limb loss [11], and has been used as a method to examine motion efficiency [56,57]. Max normalization was applied for each feature.

Understanding one of the limitations of k-means clustering to be the dependence of the output on cluster center initialization, cluster center initialization used the K-means++ algorithm (Statistics and Machine Learning Toolbox, MATLAB) with the squared Euclidean as the distance metric. To further improve consistency in results by avoiding local minima, the clustering algorithm was implemented with five replicates.

**Fig 2:**
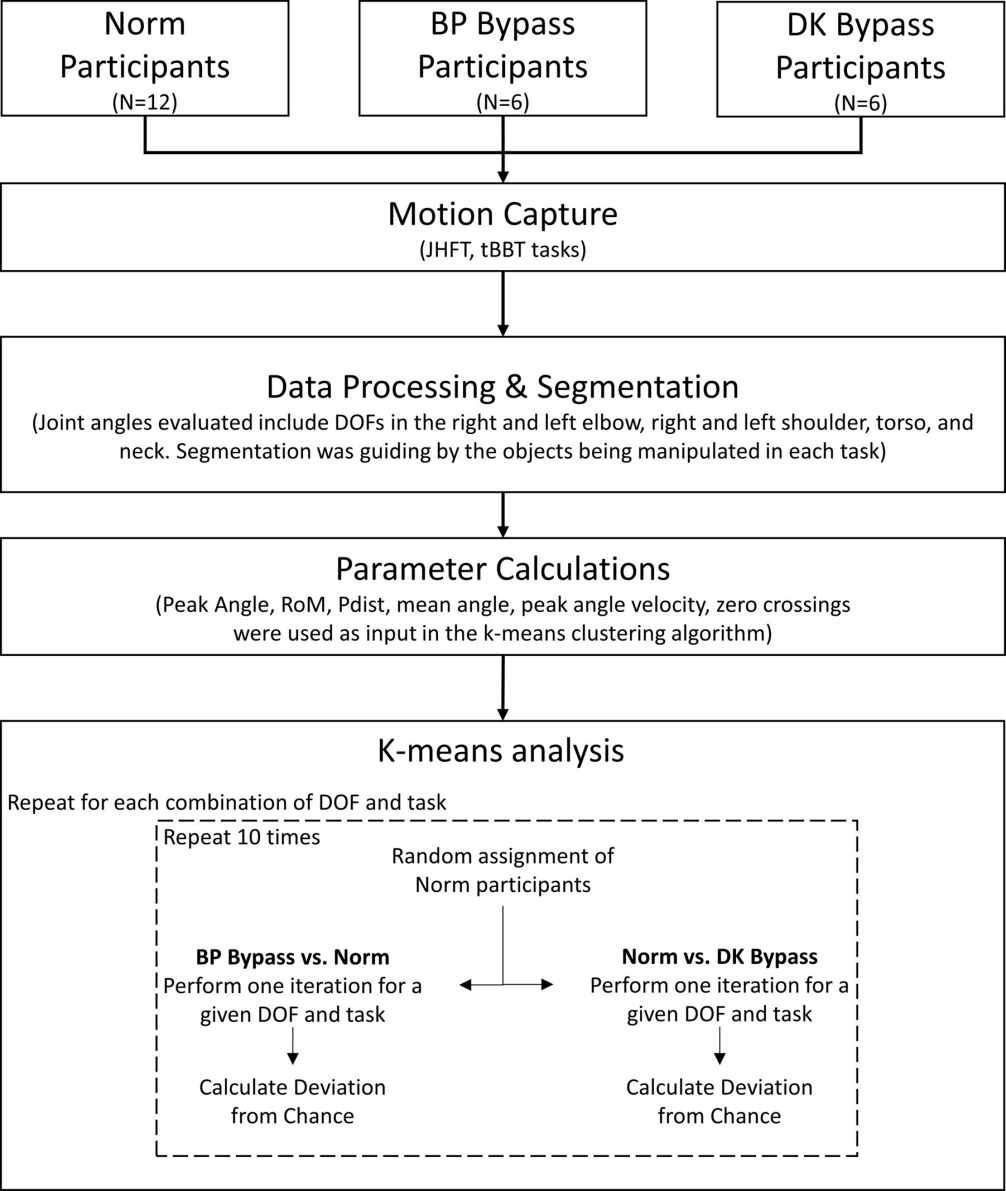
Flowchart of data collection and processing and the calculation of deviation from chance (DfC) metric.

K-means clustering analyses were performed between the normative condition data and each bypass condition data (i.e. Norm vs. BP Bypass and Norm vs. DK Bypass), for each task and DOF, resulting in a total of 126 K-means analyses for one bypass condition (9 tasks x 14 DOFs per condition). For each analysis, ten iterations were performed, with randomized Norm controls assigned for each iteration. That is, each k-means iteration for a given task and DOF had the twelve normative participants randomly assigned into either the BP Bypass control group or the DK Bypass control group. This approach was implemented to account for variation in normal movement patterns, and to provide an estimate of the classification accuracy range. A flowchart of the data collection, processing, and analysis is shown in in Fig 2. In total, 2,520 K-means were performed (9 tasks x 14 DOFs x 10 iterations x 2 conditions).

#### Evaluation of algorithm performance–Deviation from Chance (DfC) metric

Although the true category of each data point was known (i.e. Norm or Bypass), the unsupervised approach employed in this study assigns an arbitrary category to every “cluster” that is formed. Thus, when determining accuracy of the classification, a new accuracy metric was used that provides the accuracy of clustering regardless of the actual labeling of any cluster, if the data points of any given cluster are maintained as a single cluster. The new metric utilized in this paper measured the absolute difference of the accuracy from random chance, which is 50% for a binary classification. We refer to this metric as the deviation from chance or DfC (Eq 2). The DfC ranges from 0 to 50, with a result of 0 indicating that a particular DOF was not informative in distinguishing the Norm and Bypass conditions (i.e. classification accuracy matches that of random chance) and a result of 50 indicating a particular DOF was highly informative in distinguishing the Norm and Bypass conditions. A threshold for this DfC metric was selected to be 25, half of the maximum possible value, as an initial value. More or less strict requirements can be placed on the identification of DOFs that distinguish the Norm and Bypass conditions by increasing or decreasing this DfC threshold, respectively.

$$DfC=\left|Kmeans\;labeling\;accuracy\;\%-\left(\frac{100}{\#\;Labeling\;Categories}\right)\right|$$ (2)

#### Statistical analysis

To determine the DfC values for a given DOF/task that are significantly higher than the DfC threshold of 25, a one-sided Wilcoxon ranksum test was performed between the DfC values resulting from the 10 iterations for a given task/DOF and the threshold (α = 0.05). Since the goal of this work is to identify the individual DOFs and tasks that elicit significantly different movement from normative movement during use of a bypass prosthesis, an adjustment for multiple comparisons was not done. However, a table of p-values for this analysis is included in supplementary material (S1 Table).

The distributions of all features used in the k-means analyses are also plotted for each DOF and task (S1–S14 Figs). For informational purposes, a Wilcoxon ranksum test between each bypass condition and the norm condition was done with a significance level set at α = 0.05. While a comparison of clustering approaches to conventional statistical approaches is beyond the scope of this paper, these data are provided for transparency purposes.

## Results

**Fig 3:**
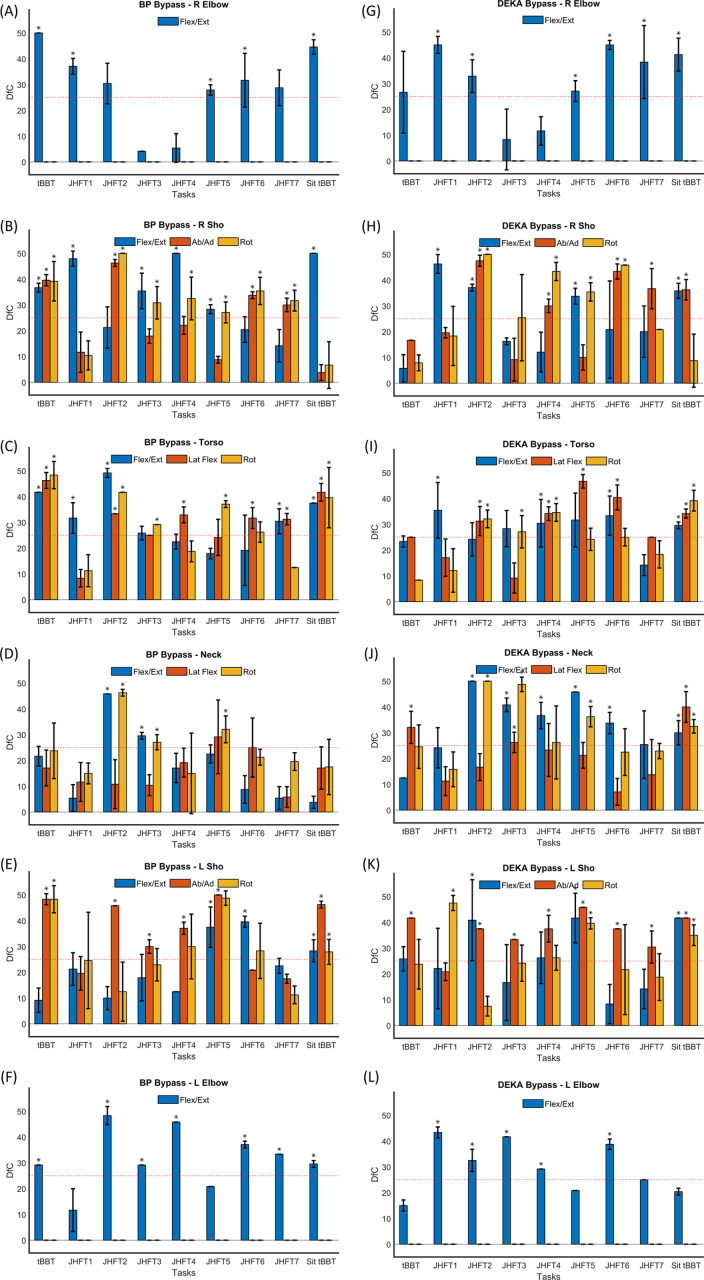
Average and standard deviation from chance (DfC) for all tasks, DOFs, and bypass condition comparisons: A-F) Body Powered Bypass v. Norm Joint results, G-L) DK Bypass v. Norm Joint Results. Tasks: JHFT1—Writing, JHFT2—Page Turning, JHFT3—Picking Up Small Objects, JHFT4—Simulated Feeding, JHFT5—Stacking Checkers, JHFT6—Moving Large Light Objects and JHFT7—Moving Large Heavy Objects. Distributions significantly greater than the threshold at DfC = 25 (red dotted line) denoted by a * (p < 0.05).

This work successfully created kinematic datasets with representative activities of daily living motions in multiple populations of simulated device users. Between the neck, torso, right and left elbows, and right and left shoulders, there were 14 DOFs analyzed for each of the 9 tasks performed. The average DfC metric from the K-means analyses are plotted for each individual joint and task, with error bars on the bar plots indicating the standard deviation of the 10 k-means iterations (Fig 3). Asterisks in the bar plots indicate that a given DOF/task for a condition resulted in a significantly higher DfC values than the threshold. This figure allows for identification of tasks that separate normative movement from bypass device movement as a function of each analyzed DOF. The threshold for DfC was denoted through the red dotted line at 25 (Fig 3). In general, the tasks that exceeded the threshold varied across the bypass conditions and the DOFs within each joint. The results were consistent with previous studies in showing that tasks and joint DOF involved in abnormal movement are device-specific. However, certain patterns did emerge and will be discussed in the Discussion section.

To get a better sense of which tasks and DOFs were associated with the greatest distinguishability between normative movement and bypass condition movement, the K-means results that were significantly greater than the DfC threshold were summarized for each bypass condition. Out of the 126 k-means analyses for the BP bypass condition (9 tasks x 14 DOFs) performed, 62 were significantly higher than the threshold (p < 0.05). For those 62 analyses, the frequency of appearance of a particular task (Table 1A) or DOF (Table 1B) was determined and presented as a percentage. The same summary was done for the DK bypass condition, with 64 out of 126 analyses significantly exceeding the threshold, and the frequency of a particular task (Table 1A) or DOF (Table 1B) was determined.

**Table 1:** Percentage of times a (A) task and (B) DOF were associated with deviations from chance distributions significantly greater than the threshold.

**(A)**	Task Frequency (%)	
**Task**	**BP Bypass**	**DEKA Bypass**
tBBT	16.1	3.1
JHFT1—write	4.8	7.8
JHFT2—page turn	14.5	17.2
JHFT3–small objects	11.3	9.4
JHFT4 –simulated feeding	8.1	12.5
JHFT5 –stacking checkers	12.9	14.1
JHFT6–light cans	9.7	12.5
JHFT7–heavy cans	8.1	4.7
Sit_tBBT	14.5	18.8
**(B)**	DOF Frequency (%)	
**DOF**	**BP Bypass**	**DEKA Bypass**
R Elbow–Flex/Ext	8.1	9.4
L Elbow–Flex/Ext	11.3	7.8
R Sho–Flex/Ext	9.7	6.3
R Sho–Ab/Ad	6.5	7.8
R Sho–Rot	11.3	6.3
L Sho–Flex/Ext	4.8	4.7
L Sho–Ab/Ad	9.7	12.5
L Sho–Rot	4.8	4.7
Neck–Flex/Ext	3.2	9.4
Neck–Lat Flex	0.0	4.7
Neck–Rot	4.8	6.3
Torso–Flex/Ext	8.1	6.3
Torso–Lat Flex	9.7	7.8
Torso–Rot	8.1	6.3

One of the most frequent tasks with high DfC values for both the BP bypass and DK bypass conditions was JHFT2 –Page Turning, occurring in 14.5% and 17.2% of the significant results, respectively (Table 1A). Some tasks were found to elicit more significant DfC values for one bypass condition, but not the other. For example, 16.1% of the significant results were elicited by tBBT in the BP bypass condition compared to only 3.1% for this same task in the DK bypass condition.

When all DOFs are combined, the right shoulder and torso appear more often in the significant results across both bypass conditions, indicating these joints are most informative for distinguishing movement between the Norm and Bypass conditions (Table 1B). Interestingly, left shoulder abduction/adduction appears often in the significant results for both the BP (9.7%) and DK (12.5%) bypass conditions. While each DOF tends to be equally represented in the significant results across bypass conditions, there is a large discrepancy in the neck DOFs, with these DOFs appearing more often when distinguishing movement between the Norm condition and DK bypass condition.

## Discussion and conclusion

The goals of this work were to utilize unsupervised machine learning to identify parameters that will inform abnormal movement for several tasks and elucidate the variations in movement approach across two different upper-limb prosthesis devices with varying DOFs as compared to non-disabled controls. To achieve those goals, specific features of movement derived from motion capture data from individuals with no upper limb impairment or disability under normative conditions and individuals using two upper limb bypass prosthesis devices (body-powered hook and DEKA arm) were used as input into a K-means clustering algorithm. While a few studies have focused on the application of clustering algorithms to the upper limb prosthesis user population, the use of these clustering methods to compare movement of individuals using these two prosthetic devices to the movements of non-disabled individuals has not been done.

Although patterns emerged, there was no single DOF that was universally distinctive across tasks and Bypass conditions (Fig 3) and no single task that consistently elicited distinctive movements between the Norm and Bypass conditions (Fig 3). This result is not unexpected, however, based on previous studies that have indicated compensation patterns for this clinical population are both task and device dependent [2,20]. The BP Bypass results, with highly distinct torso and shoulder angles, were consistent with the results from Metzger et al. 2012 which had 7 of the 10 participants using a body powered device [11]. Additionally, the compensatory motions of the head and torso found by Hussaini et al. 2017 in prosthesis users equipped with single DOF electric hand terminal devices [3] also corresponded well with the distinct joint angles identified in the BP Bypass condition. With a more robust approach to characterizing movement that simultaneously incorporates multiple features of movement into advanced clustering algorithms to identify differences, the results presented here still build on existing knowledge and can be used to inform the development of scoring methodology for upper limb performance-based outcome measures. A discussion of the clinical implications of the results as well as limitations and future work follows.

According to Table 1B, the right shoulder and torso are more frequently associated with high DfC values, meaning differences in normative and bypass prosthesis user movement are greater for these body regions. Fig 3B and 3H indicate that tasks requiring a lot of wrist motion, such as JHFT2—page turning and JHFT6 –moving large, light objects, will challenge shoulder abduction and internal rotation for both BP and DK Bypass prosthesis users, but not so much shoulder flexion. This information could be used to devise more targeted approaches for the assessment of normal movement for tasks typically requiring wrist manipulation. Instead of simply asking an observer to make a subjective assessment of the normalcy of movement, instructions could be provided to closely observe how far the upper arm goes out to the side or how much rotation is required of the upper arm to complete a task. Similarly, instructions could be provided to focus more on torso rotation as opposed to torso forward flexion or lateral flexion for tasks that require small object manipulation. Fig 3C and 3I indicate for tasks such as JHFT3 –small objects and JHFT5—stacking checkers, torso rotation is the DOF that differs most consistently between non-disabled controls and bypass prosthesis users. Results also show that left shoulder abduction/adduction is frequently different between non-disabled controls and bypass prosthesis users, as this DOF appears in 9.7% and 12.5% of results significantly higher than the threshold for the BP and DK bypass conditions, respectively (Table 1B). This is not an unexpected result for the BP bypass prosthesis since this prosthetic device type is controlled by movement of the contralateral arm. That the DK bypass condition also shows high use of this DOF may indicate the weight of the DK device causes extra counter-movements in the opposite arm. Although the experimental protocol included a weight balancer and arm brace to reduce the impact of the DK bypass weight on movement, we cannot discount the fact that this may be an artifact of the experimental design. Repeating the experiment in actual DK prosthesis users would illuminate the tendency of this user group to move the contralateral limb during unilateral tasks.

As mentioned previously, the type of device being used may also be an important factor to consider when assessing normalcy of movement and the impact of the device on compensatory movement. Fig 3B, for example, shows that right shoulder flexion/extension during JHFT4 –simulated feeding is significantly different between non-disabled control movement and BP Bypass prosthesis user movement. However, Fig 3H shows that right shoulder abduction/adduction and rotation are the DOFs that distinguish non-disabled control movement from DK Bypass prosthesis user movement for this same task. Another interesting observation showing the potential impact of device on movement can be seen in the neck DOFs in Table 1B and DfC plots in Fig 3D and 3J. Of all the DOFs that appeared in the results significantly higher than the DfC threshold of 25 for the BP bypass condition, only 8.1% included neck DOFs. Conversely, 20.3% of the significant results for the DK bypass included neck DOFs. This implies neck movements of individuals using the DK bypass prosthesis are more often different from the non-disabled control group. The result can be explained by considering the visibility of objects being manipulated by the terminal device of these two prosthetic systems, with body-powered hooks more typically known for good visibility of objects [58]. Understanding how the device impacts the movement of the user is important to assessing the normalcy of the observed movement, and the results presented here serve as a preliminary source for elucidating such an impact for two types of upper limb prosthetic devices.

Due to the binary nature of the classification problem (i.e. Norm vs a Bypass), and the simplicity of the algorithm’s decision-making approach, K-means clustering was selected for this study. K-means clustering is a partitioning algorithm that divides data into groups of at least one value with each data value assigned to exactly one group, by minimizing the mean distance of the data value from an assigned cluster center value in repeated rounds of reassignment as necessary [59]. Alternate partitioning algorithms, model-based algorithms, or density-based clustering algorithms may provide greater separation between conditions. The features selected (peak angle, range of motion, path distance, mean angle, peak angle velocity, and zero crossings) were selected for their ease in translation to actual motion and understandability. This study aimed to use an easily understandable machine learning approach to examine motion data for useful differences, so no dimensionality reduction approaches were applied. The identification of features most informative to the difference between normative and bypass prosthesis user movement was out of scope for this paper, but such an analysis may help contribute to consensus on the definition of movement quality.

One output of this work is the creation of kinematic datasets with representative ADL motions in multiple populations of simulated device users. Even with only six features characterizing movement, the K-means clustering approach was able to identify distinctive joint DOFs for several ADL tasks over two methods of simulated upper limb prosthesis use. With the development of more compact, easily implementable motion analysis systems such as 3D optical marker-based motion capture, inertial measurements units, or markerless motion capture that can be used in the home or clinic, the widespread collection of quantitative movement data is becoming more of a reality. Although limited in generalizability to actual prosthesis users, this dataset can be used to inform the development of larger, more applicable datasets to be incorporated into a supervised machine learning algorithm for the real-time identification of abnormal or compensatory movement based on movement data input.

Key limitations of this study include the limited sample size due to the high training requirements for skillful bypass device use, the use of non-disabled participants, the unilateral nature of the tasks selected, as well as the limited interaction zones required for task performance. While the use of uniformly trained non-disabled participants limits the sources of kinematic variation to those of the device and potentially allows for a larger participant pool, the generalizability of these results to individuals with upper limb loss remains to be seen. Furthermore, the unilateral nature of the tasks selected allowed the examination of device induced changes in kinematics but may not be fully representative of device use patterns outside of the lab environment. Lastly, all tasks were performed in a frontal central interaction zone, which reflects the task space of performance-based outcome measure tasks in common use [47]. However, device induced kinematic changes outside of this interaction zone were not captured.

Future avenues of investigation include increasing the number of trained participants, comparing the results from this study with the actual upper limb prosthesis user motion capture data, investigating additional features for machine learning, investigating additional clustering approaches, and performing further experiments with additional tasks that require device use in spaces other than directly in front of the participant.

## Supporting information

S1 Fig Distributions of features for DEKA Bypass (blue), BP Bypass (red), and Norm (black) conditions for right elbow flexion/extension. Tasks JHFT1 -Writing, JHFT2 -Page Turning, JHFT3 -Picking Up Small Objects, JHFT4 -Simulated Feeding, JHFT5 -Stacking Checkers, JHFT6 -Moving Large Light Objects and JHFT7 -Moving Large Heavy. Stars denote statistical significance of Wilcoxon ranksumtest between each bypass condition data and the Norm condition data. *p< 0.05, **p<0.01, ***p<0.001.
(PDF)

S2 Fig Distributions of features for DEKA Bypass (blue), BP Bypass (red), and Norm (black) conditions for right shoulder flexion/extension. Tasks JHFT1 -Writing, JHFT2 -Page Turning, JHFT3 -Picking Up Small Objects, JHFT4 -Simulated Feeding, JHFT5 -Stacking Checkers, JHFT6 -Moving Large Light Objects and JHFT7 -Moving Large Heavy. Stars denote statistical significance of Wilcoxon ranksumtest between each bypass condition data and the Norm condition data. *p< 0.05, **p<0.01, ***p<0.001.
(PDF)

S3 Fig Distributions of features for DEKA Bypass (blue), BP Bypass (red), and Norm (black) conditions for right shoulder abduction/adduction. Tasks JHFT1 -Writing, JHFT2 -Page Turning, JHFT3 -Picking Up Small Objects, JHFT4 -Simulated Feeding, JHFT5 -Stacking Checkers, JHFT6 -Moving Large Light Objects and JHFT7 -Moving Large Heavy. Stars denote statistical significance of Wilcoxon ranksumtest between each bypass condition data and the Norm condition data. *p< 0.05, **p<0.01, ***p<0.001.
(PDF)

S4 Fig Distributions of features for DEKA Bypass (blue), BP Bypass (red), and Norm (black) conditions for right shoulder rotation. Tasks JHFT1 -Writing, JHFT2 -Page Turning, JHFT3 -Picking Up Small Objects, JHFT4 -Simulated Feeding, JHFT5 -Stacking Checkers, JHFT6 -Moving Large Light Objects and JHFT7 -Moving Large Heavy. Stars denote statistical significance of Wilcoxon ranksumtest between each bypass condition data and the Norm condition data. *p< 0.05, **p<0.01, ***p<0.001.
(PDF)

S5 Fig Distributions of features for DEKA Bypass (blue), BP Bypass (red), and Norm (black) conditions for torso flexion. Tasks JHFT1 -Writing, JHFT2 -Page Turning, JHFT3 -Picking Up Small Objects, JHFT4 -Simulated Feeding, JHFT5 -Stacking Checkers, JHFT6 -Moving Large Light Objects and JHFT7 -Moving Large Heavy. Stars denote statistical significance of Wilcoxon ranksumtest between each bypass condition data and the Norm condition data. *p< 0.05, **p<0.01, ***p<0.001.
(PDF)

S6 Fig Distributions of features for DEKA Bypass (blue), BP Bypass (red), and Norm (black) conditions for torso lateral flexion. Tasks JHFT1 -Writing, JHFT2 -Page Turning, JHFT3 -Picking Up Small Objects, JHFT4 -Simulated Feeding, JHFT5 -Stacking Checkers, JHFT6 -Moving Large Light Objects and JHFT7 -Moving Large Heavy. Stars denote statistical significance of Wilcoxon ranksumtest between each bypass condition data and the Norm condition data. *p< 0.05, **p<0.01, ***p<0.001.
(PDF)

S7 Fig Distributions of features for DEKA Bypass (blue), BP Bypass (red), and Norm (black) conditions for torso rotation. Tasks JHFT1 -Writing, JHFT2 -Page Turning, JHFT3 -Picking Up Small Objects, JHFT4 -Simulated Feeding, JHFT5 -Stacking Checkers, JHFT6 -Moving Large Light Objects and JHFT7 -Moving Large Heavy. Stars denote statistical significance of Wilcoxon ranksumtest between each bypass condition data and the Norm condition data. *p< 0.05, **p<0.01, ***p<0.001.
(PDF)

S8 Fig Distributions of features for DEKA Bypass (blue), BP Bypass (red), and Norm (black) conditions for neck flexion. Tasks JHFT1 -Writing, JHFT2 -Page Turning, JHFT3 -Picking Up Small Objects, JHFT4 -Simulated Feeding, JHFT5 -Stacking Checkers, JHFT6 -Moving Large Light Objects and JHFT7 -Moving Large Heavy. Stars denote statistical significance of Wilcoxon ranksumtest between each bypass condition data and the Norm condition data. *p< 0.05, **p<0.01, ***p<0.001.
(PDF)

S9 Fig Distributions of features for DEKA Bypass (blue), BP Bypass (red), and Norm (black) conditions for neck lateral flexion. Tasks JHFT1 -Writing, JHFT2 -Page Turning, JHFT3 -Picking Up Small Objects, JHFT4 -Simulated Feeding, JHFT5 -Stacking Checkers, JHFT6 -Moving Large Light Objects and JHFT7 -Moving Large Heavy. Stars denote statistical significance of Wilcoxon ranksumtest between each bypass condition data and the Norm condition data. *p< 0.05, **p<0.01, ***p<0.001.
(PDF)

S10 Fig Distributions of features for DEKA Bypass (blue), BP Bypass (red), and Norm (black) conditions for neck rotation. Tasks JHFT1 -Writing, JHFT2 -Page Turning, JHFT3 -Picking Up Small Objects, JHFT4 -Simulated Feeding, JHFT5 -Stacking Checkers, JHFT6 -Moving Large Light Objects and JHFT7 -Moving Large Heavy. Stars denote statistical significance of Wilcoxon ranksumtest between each bypass condition data and the Norm condition data. *p< 0.05, **p<0.01, ***p<0.001.
(PDF)

S11 Fig Distributions of features for DEKA Bypass (blue), BP Bypass (red), and Norm (black) conditions for left shoulder flexion/extension. Tasks JHFT1 -Writing, JHFT2 -Page Turning, JHFT3 -Picking Up Small Objects, JHFT4 -Simulated Feeding, JHFT5 -Stacking Checkers, JHFT6 -Moving Large Light Objects and JHFT7 -Moving Large Heavy. Stars denote statistical significance of Wilcoxon ranksumtest between each bypass condition data and the Norm condition data. *p< 0.05, **p<0.01, ***p<0.001.
(PDF)

S12 Fig Distributions of features for DEKA Bypass (blue), BP Bypass (red), and Norm (black) conditions for left shoulder abduction/adduction. Tasks JHFT1 -Writing, JHFT2 -Page Turning, JHFT3 -Picking Up Small Objects, JHFT4 -Simulated Feeding,JHFT5 -Stacking Checkers, JHFT6 -Moving Large Light Objects and JHFT7 -Moving Large Heavy. Stars denote statistical significance of Wilcoxon ranksumtest between each bypass condition data and the Norm condition data. *p< 0.05, **p<0.01, ***p<0.001.
(PDF)

S13 Fig Distributions of features for DEKA Bypass (blue), BP Bypass (red), and Norm (black) conditions for left shoulder rotation. Tasks JHFT1 -Writing, JHFT2 -Page Turning, JHFT3 -Picking Up Small Objects, JHFT4 -Simulated Feeding, JHFT5 -Stacking Checkers, JHFT6 -Moving Large Light Objects and JHFT7 -Moving Large Heavy. Stars denote statistical significance of Wilcoxon ranksumtest between each bypass condition data and the Norm condition data. *p< 0.05, **p<0.01, ***p<0.001.
(PDF)

S14 Fig Distributions of features for DEKA Bypass (blue), BP Bypass (red), and Norm (black) conditions for left elbow flexion/extension. Tasks JHFT1 -Writing, JHFT2 -Page Turning, JHFT3 -Picking Up Small Objects, JHFT4 -Simulated Feeding, JHFT5 -Stacking Checkers, JHFT6 -Moving Large Light Objects and JHFT7 -Moving Large Heavy. Stars denote statistical significance of Wilcoxon ranksumtest between each bypass condition data and the Norm condition data. *p< 0.05, **p<0.01, ***p<0.001.
(PDF)

S1 Table P-values for the statistical test comparing the Deviation from Chance (DfC) distributions for a given task/DOF to the threshold value (DfC = 25). (DOCX)
