# Supplementary material for: Application of machine learning to the identification of joint degrees of freedom involved in abnormal movement during upper limb prosthesis use
Source: PLoS One. 2021 Feb 11;16(2):e0246795. doi: 10.1371/journal.pone.0246795 (PMC7877744; doi:10.1371/journal.pone.0246795)
Supplement: S6 Fig — Tasks JHFT1 -Writing, JHFT2 -Page Turning, JHFT3 -Picking Up Small Objects, JHFT4 -Simulated Feeding, JHFT5 -Stacking Checkers, JHFT6 -Moving Large Light Objects and JHFT7 -Moving Large Heavy. Stars denote statistical significance of Wilcoxon ranksumtest between each bypass condition data and the Norm condition data. *p< 0.05, **p<0.01, ***p<0.001. (PDF) [file pone.0246795.s006.pdf]

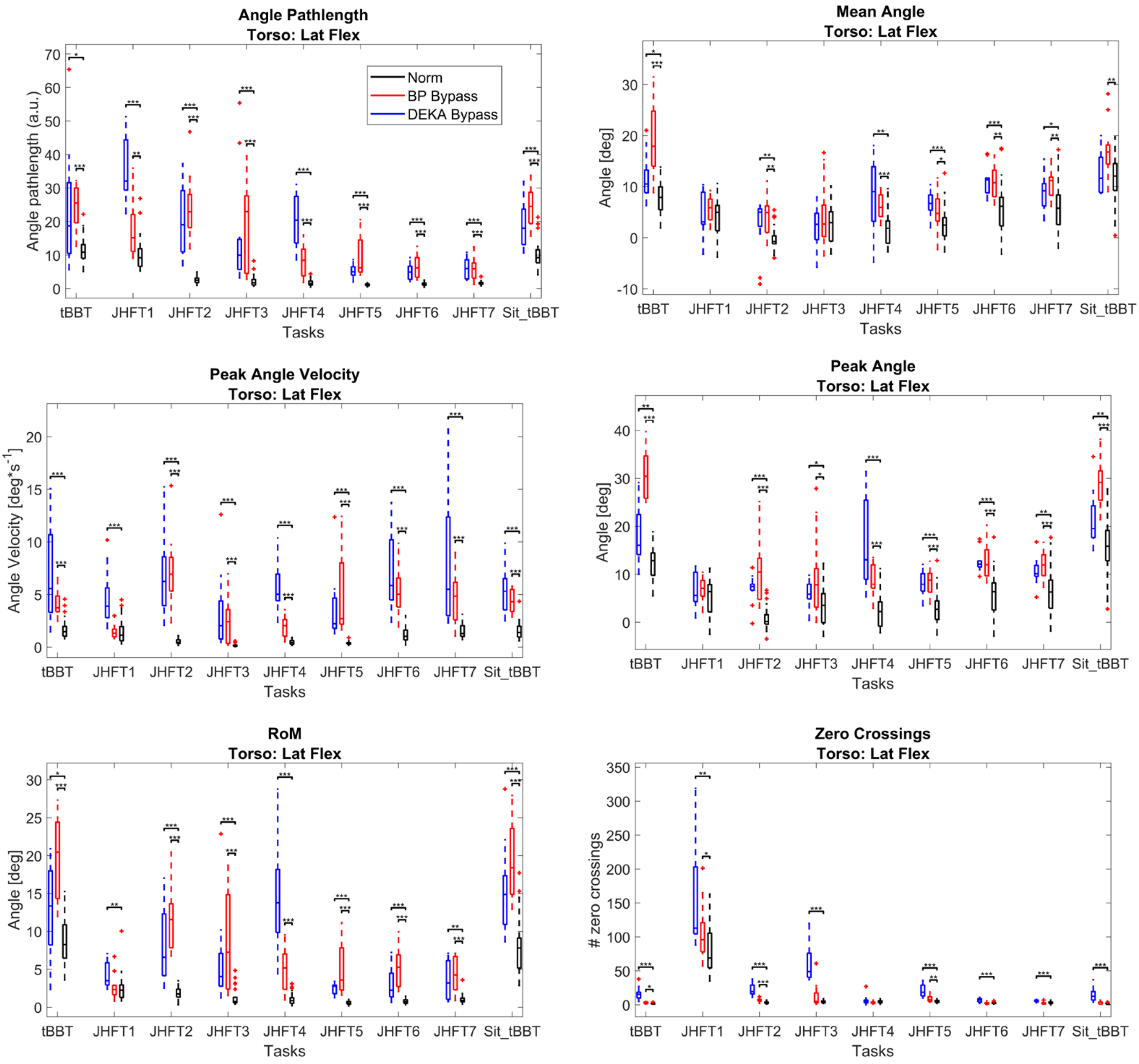

Supplemental Figure 6: Distributions of features for DEKA Bypass (blue), BP Bypass (red), and Norm (black) conditions for torso lateral flexion. Tasks JHFT1 - Writing, JHFT2 - Page Turning, JHFT3 - Picking Up Small Objects, JHFT4 - Simulated Feeding, JHFT5 - Stacking Checkers, JHFT6 - Moving Large Light Objects and JHFT7 - Moving Large Heavy. Stars denote statistical significance of Wilcoxon ranksum test between each bypass condition data and the Norm condition data. *p< 0.05, **p<0.01, ***p<0.001.
