# Supplementary material for: Application of machine learning to the identification of joint degrees of freedom involved in abnormal movement during upper limb prosthesis use
Source: PLoS One. 2021 Feb 11;16(2):e0246795. doi: 10.1371/journal.pone.0246795 (PMC7877744; doi:10.1371/journal.pone.0246795)
Supplement: S1 Table — (DOCX) [file pone.0246795.s015.docx]

Supplementary Table 1: P-values for the statistical test comparing the Deviation from Chance (DfC) distributions for a given task/DOF to the threshold value (DfC = 25).

| **Task** | **DOF** | **BP Bypass (p-value)** | **DK Bypass (p-value)** |
| --- | --- | --- | --- |
| **tBBT** | R Elbow - Flex/Ext | 7.97E-06 | 0.219 |
|  | L Elbow - Flex/Ext | 1.64E-05 | 1.000 |
|  | R Sho - Flex/Ext | 1.64E-05 | 1.000 |
|  | R Sho - Ab/Ad | 2.65E-05 | 1.000 |
|  | R Sho - Rot | 3.08E-05 | 1.000 |
|  | L Sho - Flex/Ext | 1.000 | 0.519 |
|  | L Sho - Ab/Ad | 2.42E-05 | 2.37E-05 |
|  | L Sho - Rot | 1.21E-05 | 0.803 |
|  | Neck - Flex/Ext | 0.994 | 1.000 |
|  | Neck - Lat Flex | 0.999 | 0.001 |
|  | Neck - Rot | 0.220 | 0.355 |
|  | Torso - Flex/Ext | 2.02E-05 | 0.987 |
|  | Torso - Lat Flex | 2.86E-05 | 1.000 |
|  | Torso - Rot | 1.21E-05 | 1.000 |
| **JHFT1 - writing** | R Elbow - Flex/Ext | 2.67E-05 | 2.86E-05 |
|  | L Elbow - Flex/Ext | 1.000 | 2.55E-05 |
|  | R Sho - Flex/Ext | 2.44E-05 | 2.86E-05 |
|  | R Sho - Ab/Ad | 0.999 | 1.000 |
|  | R Sho - Rot | 1.000 | 0.960 |
|  | L Sho - Flex/Ext | 0.983 | 0.901 |
|  | L Sho - Ab/Ad | 0.983 | 0.999 |
|  | L Sho - Rot | 0.221 | 2.67E-05 |
|  | Neck - Flex/Ext | 1.000 | 0.687 |
|  | Neck - Lat Flex | 1.000 | 1.000 |
|  | Neck - Rot | 1.000 | 1.000 |
|  | Torso - Flex/Ext | 0.001 | 0.001 |
|  | Torso - Lat Flex | 1.000 | 0.998 |
|  | Torso - Rot | 1.000 | 1.000 |
| **JHFT2 - page turning** | R Elbow - Flex/Ext | 0.099 | 0.002 |
|  | L Elbow - Flex/Ext | 1.64E-05 | 0.000 |
|  | R Sho - Flex/Ext | 0.836 | 1.21E-05 |
|  | R Sho - Ab/Ad | 2.38E-05 | 2.73E-05 |
|  | R Sho - Rot | 7.97E-06 | 7.97E-06 |
|  | L Sho - Flex/Ext | 1.000 | 0.008 |
|  | L Sho - Ab/Ad | 2.37E-05 | 7.97E-06 |
|  | L Sho - Rot | 0.999 | 1.000 |
|  | Neck - Flex/Ext | 2.02E-05 | 7.97E-06 |
|  | Neck - Lat Flex | 1.000 | 1.000 |
|  | Neck - Rot | 2.55E-05 | 7.97E-06 |
|  | Torso - Flex/Ext | 1.64E-05 | 0.989 |
|  | Torso - Lat Flex | 1.21E-05 | 0.001 |
|  | Torso - Rot | 2.28E-05 | 3.03E-05 |
| **JHFT3 - small objects** | R Elbow - Flex/Ext | 1.000 | 1.000 |
|  | L Elbow - Flex/Ext | 2.02E-05 | 1.64E-05 |
|  | R Sho - Flex/Ext | 2.73E-05 | 1.000 |
|  | R Sho - Ab/Ad | 1.000 | 1.000 |
|  | R Sho - Rot | 0.015 | 0.806 |
|  | L Sho - Flex/Ext | 0.998 | 0.804 |
|  | L Sho - Ab/Ad | 2.07E-05 | 2.28E-05 |
|  | L Sho - Rot | 0.960 | 0.960 |
|  | Neck - Flex/Ext | 1.66E-05 | 2.55E-05 |
|  | Neck - Lat Flex | 1.000 | 0.034 |
|  | Neck - Rot | 0.014 | 1.66E-05 |
|  | Torso - Flex/Ext | 0.184 | 0.084 |
|  | Torso - Lat Flex | 1.000 | 1.000 |
|  | Torso - Rot | 2.28E-05 | 0.021 |
| **JHFT4 - simulated feeding** | R Elbow - Flex/Ext | 1.000 | 1.000 |
|  | L Elbow - Flex/Ext | 1.64E-05 | 2.37E-05 |
|  | R Sho - Flex/Ext | 7.97E-06 | 1.000 |
|  | R Sho - Ab/Ad | 0.994 | 0.000 |
|  | R Sho - Rot | 0.009 | 2.65E-05 |
|  | L Sho - Flex/Ext | 1.000 | 0.355 |
|  | L Sho - Ab/Ad | 2.09E-05 | 3.06E-05 |
|  | L Sho - Rot | 0.221 | 0.099 |
|  | Neck - Flex/Ext | 1.000 | 3.08E-05 |
|  | Neck - Lat Flex | 0.999 | 0.517 |
|  | Neck - Rot | 0.993 | 0.114 |
|  | Torso - Flex/Ext | 0.994 | 0.001 |
|  | Torso - Lat Flex | 3.03E-05 | 2.55E-05 |
|  | Torso - Rot | 1.000 | 2.73E-05 |
| **JHFT5 - stacking checkers** | R Elbow - Flex/Ext | 0.001 | 0.005 |
|  | L Elbow - Flex/Ext | 1.000 | 1.000 |
|  | R Sho - Flex/Ext | 0.000 | 2.88E-05 |
|  | R Sho - Ab/Ad | 1.000 | 1.000 |
|  | R Sho - Rot | 0.047 | 2.73E-05 |
|  | L Sho - Flex/Ext | 0.000 | 0.001 |
|  | L Sho - Ab/Ad | 7.97E-06 | 2.28E-05 |
|  | L Sho - Rot | 1.66E-05 | 2.37E-05 |
|  | Neck - Flex/Ext | 0.987 | 2.28E-05 |
|  | Neck - Lat Flex | 0.221 | 0.988 |
|  | Neck - Rot | 0.001 | 2.92E-05 |
|  | Torso - Flex/Ext | 1.000 | 0.057 |
|  | Torso - Lat Flex | 0.994 | 2.86E-05 |
|  | Torso - Rot | 1.21E-05 | 0.522 |
| **JHFT6 - light cans** | R Elbow - Flex/Ext | 0.021 | 2.67E-05 |
|  | L Elbow - Flex/Ext | 1.21E-05 | 2.07E-05 |
|  | R Sho - Flex/Ext | 0.996 | 0.676 |
|  | R Sho - Ab/Ad | 2.38E-05 | 2.92E-05 |
|  | R Sho - Rot | 3.10E-05 | 1.21E-05 |
|  | L Sho - Flex/Ext | 2.55E-05 | 1.000 |
|  | L Sho - Ab/Ad | 1.000 | 7.97E-06 |
|  | L Sho - Rot | 0.345 | 0.951 |
|  | Neck - Flex/Ext | 1.000 | 0.000 |
|  | Neck - Lat Flex | 0.355 | 1.000 |
|  | Neck - Rot | 0.999 | 0.815 |
|  | Torso - Flex/Ext | 0.951 | 0.006 |
|  | Torso - Lat Flex | 2.86E-05 | 2.09E-05 |
|  | Torso - Rot | 0.184 | 0.519 |
| **JHFT7 - heavy cans** | R Elbow - Flex/Ext | 0.115 | 0.001 |
|  | L Elbow - Flex/Ext | 2.02E-05 | 1.000 |
|  | R Sho - Flex/Ext | 1.000 | 0.901 |
|  | R Sho - Ab/Ad | 0.000 | 0.001 |
|  | R Sho - Rot | 0.000 | 1.000 |
|  | L Sho - Flex/Ext | 0.994 | 1.000 |
|  | L Sho - Ab/Ad | 1.000 | 0.007 |
|  | L Sho - Rot | 1.000 | 0.952 |
|  | Neck - Flex/Ext | 1.000 | 0.221 |
|  | Neck - Lat Flex | 1.000 | 0.998 |
|  | Neck - Rot | 1.000 | 0.987 |
|  | Torso - Flex/Ext | 0.002 | 1.000 |
|  | Torso - Lat Flex | 2.97E-05 | 1.000 |
|  | Torso - Rot | 1.000 | 1.000 |
| **Sit tBBT** | R Elbow - Flex/Ext | 2.67E-05 | 3.08E-05 |
|  | L Elbow - Flex/Ext | 2.55E-05 | 1.000 |
|  | R Sho - Flex/Ext | 7.97E-06 | 2.09E-05 |
|  | R Sho - Ab/Ad | 1.000 | 2.88E-05 |
|  | R Sho - Rot | 1.000 | 0.998 |
|  | L Sho - Flex/Ext | 0.015 | 2.28E-05 |
|  | L Sho - Ab/Ad | 2.07E-05 | 2.37E-05 |
|  | L Sho - Rot | 0.048 | 2.92E-05 |
|  | Neck - Flex/Ext | 1.000 | 0.002 |
|  | Neck - Lat Flex | 0.993 | 3.06E-05 |
|  | Neck - Rot | 0.988 | 7.82E-05 |
|  | Torso - Flex/Ext | 7.97E-06 | 2.55E-05 |
|  | Torso - Lat Flex | 2.99E-05 | 2.65E-05 |
|  | Torso - Rot | 0.001 | 2.71E-05 |
